# A Bioinspired Gelatin–Amorphous Calcium Phosphate Coating on Titanium Implant for Bone Regeneration

**DOI:** 10.1002/adhm.202203411

**Published:** 2023-04-02

**Authors:** Yanhuizhi Feng, Di Wu, Jennifer Knaus, Sascha Keßler, Bing Ni, ZongKun Chen, Johnathan Avaro, Rui Xiong, Helmut Cölfen, Zuolin Wang

**Affiliations:** ^1^ Department of Implantology Stomatological Hospital and Dental School of Tongji University Shanghai Engineering Research Center of Tooth Restoration and Regeneration 200072 Shanghai China; ^2^ Department of Chemistry Physical Chemistry University of Konstanz Universitätsstrasse 10 78457 Konstanz Germany; ^3^ EMPA Material and Science Technology Lerchenfeldstrasse 5 9014 St. Gallen Switzerland

**Keywords:** bioinspired materials, gelatin‐CaPO_4_ coatings, mineralization, osseointegration, titanium implants

## Abstract

Biocompatible and bio‐active coatings can enhance and accelerate osseointegration via chemical binding onto substrates. Amorphous calcium phosphate (ACP) has been shown as a precursor to achieve mineralization in vertebrates and invertebrates under the control of biological macromolecules. This work presents a simple bioinspired Gelatin‐CaPO_4_ (Gel‐CaP) composite coating on titanium surfaces to improve osseointegration. The covalently bound Gel‐CaP composite is characterized as an ACP‐Gel compound via SEM, FT‐IR, XRD, and HR‐TEM. The amorphous compound coating exhibits a nanometer range thickness and improved elastic modulus, good wettability, and nanometric roughness. The amount of grafted carboxyl groups and theoretical thickness of the coatings are also investigated. More importantly, MC3T3 cells, an osteoblast cell line, show excellent cell proliferation and adhesion on the Gel‐CaP coating. The level of osteogenic genes is considerably upregulated on Ti with Gel‐CaP coatings compared to uncoated Ti, demonstrating that Gel‐CaP coatings possess a unique osteogenic ability. To conclude, this work offers a new perspective on functional, bioactive titanium coatings, and Gel‐CaP composites can be a low‐cost and promising candidate in bone regeneration.

## Graphical abstract

A covalently binding gelatin‐amorphous calcium phosphate composite coating has been successfully fabricated on titanium by biomineralization. The Gel‐ACP coatings can release calcium ions and exert favorable nano‐topography, good surface roughness, and high wettability, further promoting cell adhesion and osteoblasts differentiation. Gel‐ACP coating can provide a new perspective for titanium implant modification.

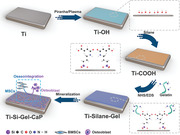

## 1 Introduction

Dental titanium implants have been in use in clinical applications for more than 40 years due to their remarkable mechanical properties and bioinert ability compared with other artificial materials. Despite the many advantageous properties, initial osseointegration and fast healing of bone implants are still important issues in the clinical field. Numerous studies showed that surface composition and topography could play important roles in the ingrowth of such implants and could improve the cell‐implant interaction.^[^ ^1^ ^]^ Generally, depending on the bio‐response of the body, implants can be categorized into several groups: bio‐tolerant, bio‐inert, and bio‐reactive.^[^ ^2^ ^]^ Since the surface properties of the implant primarily govern the biological response to implants, it is critical to engineer the surface of the implants appropriately to achieve the desired surface interaction with the surrounding cells and proteins.^[^ ^3^ ^]^ Since titanium metal is bio‐inert, Ti implants have been designed with bioactive oxide surfaces to promote osteoblast adhesion and proliferation, leading to active bone formation.^[^ ^4^ ^]^

In general, the methods for titanium surface modification can be divided into different strategies: a physical treatment and chemical techniques or combinations thereof. Commercial treatments like sandblasting, grit blasting, and ion beam‐assisted deposition have been carried out to increase the surface roughness to enhance positive cell behavior.^[^ ^5^ ^]^ Abundant studies showed that surface roughness and surface wettability could influence biomechanical fixation and osteogenic cell adhesion, differentiation, proliferation, and calcification.^[^ ^6^ ^]^ However, physically treated implants, e.g., sandblasted materials, could pose potential risks of surface corrosion or surface contamination due to the presence of blasting particle remnants.^[^ ^7^ ^]^ Such particles could induce inflammation at the bone‐implant interface and induce osteoclast activity. Unfortunately, these modification techniques demand expensive and massive instruments to operate. Due to chemical modifications, such as alkali heat treatment or electrochemical anodization, activation of the titanium surface and resulting attraction of osteoblast cells could be achieved.^[^ ^8^ ^]^ Especially, oxygen plasma is one of the simple procedures to alter titanium surface chemistry and wettability.^[^ ^9^ ^]^ Chemical modifications can be feasible for surface modifications with their cheap cost and simple procedures.

An ideal coating should not only mimic the bone structure and establish a fast bonding to the host bone, enabling fast healing and integration. In recent decades, calcium phosphate (Ca‐P) coatings have become a promising alternative to enhance metallic implants’ osteointegration and improve stress shielding.^[^ ^10^ ^]^ Hydroxyapatite (HAP), which could exhibit physiological compatibility and osteoconductive properties, became the first choice of titanium coatings. Besides, it was assumed that nano‐dimensional HAP particles have a higher surface area and play an essential role in facilitating favorable osteogenic cell adhesion and proliferation.^[^ ^11^ ^]^ Nanometric calcium phosphate like Calcium‐deficient hydroxyapatite (CDHAP) with needle shape could generate a favorable osteoimmune environment to regulate osteoblast differentiation and osteogenesis.^[^ ^12^ ^]^ However, the problem with physically bound coatings is their partial or complete detachment by particle abrasion or coating delamination, which could lead to inflammation.^[^ ^13^ ^]^

Amorphous calcium phosphate (ACP) is another phase in the group of calcium orthophosphates and was reported to be more beneficial for promoting early bone formation and remineralization than highly crystalline HAP.^[^ ^14^ ^]^ From the “bio‐reactivity” point of view, ACP could facilitate bone‐like apatite development more efficiently, thus inducing faster bone regeneration.^[^ ^15^ ^]^ It is reported that ACP and HAP coatings would not be dissolved in SBF solution, thus not weakening the bonding strength and decreasing the adhesive strength.^[^ ^16^ ^]^

From the viewpoint of bone formation, many studies supported the theory of ACP precursors in bone mineralization. They showed a disordered ACP as a major component in the newly formed parts of the zebrafish fin bone. Observation in invertebrates also illustrated that the initially deposited ACP transforms into a crystalline mineral phase over time via deposition inside the gap regions of collagen fibrils directly or delivering as extra fibrillar nanoparticles.^[^ ^17^ ^]^ However, this is still disputed for vertebrates since an amorphous phase is hard to detect or observe in the formation of bone minerals by conventional analytical techniques. Nevertheless, recent studies established a successful biomimetic mineralization model in vitro.^[^ ^18^ ^]^ For example, Andersson et al. offered a possible detailed transformation mechanism of amorphous calcium phosphate spherical particles to apatite platelet‐like crystals.^[^ ^19^ ^]^ All these studies point out the significance of ACP in organism mineralization.

In nature, macromolecules are known to serve as a template to control the growth of calcium phosphate crystals. Imai et al. reported that collagen‐derived gelatin molecules could govern the construction of lattice architecture of dicalcium phosphate.^[^ ^20^ ^]^ Sommerdijk et al. also demonstrated that collagen promotes the infiltration of ACP into fibers to control mineralization actively by exploiting polyaspartic acid (pAsp) as an inhibitor.^[^ ^21^ ^]^ Tay et al. showed that the same is possible using a polycation and pointed out the role of a Gibbs‐Donnan equilibrium for collagen intrafibrillar mineralization.^[^ ^22^ ^]^ A model of “brick‐and‐mortar” nacre explained the relationship of ACP, HAP, and biological molecules during the process of aggregation of nanometric apatite. ACP and macromolecules could act as “mortar” to cement the crystallized “bricks” of HAP.^[^ ^23^ ^]^ In order to improve the compressive strength of calcium phosphate cement, up to 10.7 – 14 Mpa, Panzavolta et al. showed that the addition of gelatin to calcium phosphate could improve the compressive strength up to 10.7 – 14 Mpa, compared to 2 – 4 Mpa.^[^ ^24^ ^]^ Thus, gelatin‐CaP composites appear to be a promising material with excellent mechanical properties as a coating for a bone implant.

A noncovalent gelatin coating of arterial implants was already reported^[^ ^25^ ^]^ as well as noncovalent titanium implant collagen coating,^[^ ^26^ ^]^ also in CaP‐mineralized form of collagen and gelatin.^[^ ^27^ ^]^ However, to prevent potential detachment, which is problematic for physically bound coatings, forming covalent bonds between implant and coatings is an advantageous strategy.^[^ ^13^ ^]^ This has indeed already been realized by covalently binding hyaluronan to titanium^[^ ^28^ ^]^ as well as collagen type 1 via silane chemistry.^[^ ^29^ ^]^ However, as gelatin is much better water‐soluble than collagen and cheaper, we adapt similar chemistry to gelatin towards a low‐cost strategy to endow titanium implants with a bioactive property to achieve stable initial osteointegration and rapid bone formation. We fabricated the titanium implant surface coatings by grafting two different silane coupling agents through vapor and liquid methods. Then, in vitro mimicking biomineralization was introduced in synergy with a biocompatible HAP nucleation inhibitor (i.e., pAsp). The underlying mechanism of mineralization has also been investigated to confirm if the compound is similar to a bone apatite precursor. More importantly, the osteoblast response stimulated by the Gel‐CaP coating in vitro was evaluated showing high expression of osteogenic genes in both different Gel‐CaP coatings.

## 2 Results and Discussion

### 2.1 Morphologies and Elemental Distribution within the Coatings

**Figure 1:**
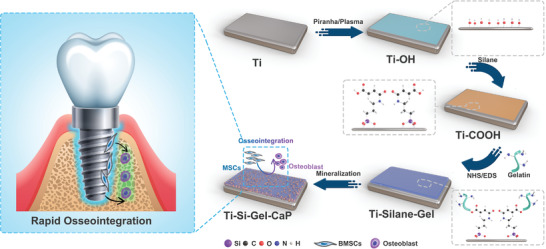
Schematic illustration of the Gel‐CaP coating formation on Ti surface.

**Figure 2:**
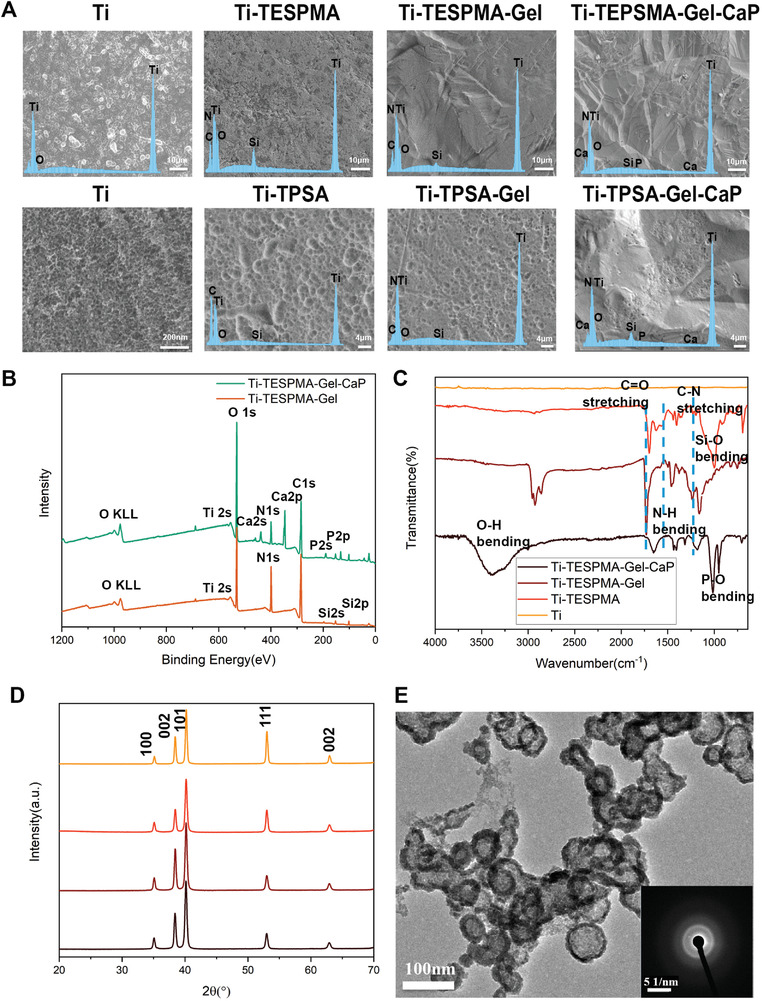
A) Surface SEM images and EDS pattern for the seven different compounds. B) XPS survey spectra of Ti‐TESPMA‐Gel and Ti‐TESPMA‐Gel‐CaP, C) FT‐IR spectra analysis of Ti‐TESPMA‐Gel‐CaP, Ti‐TESPMA‐Gel, Ti‐TESPMA, and Ti samples, D) XRD spectra of Ti‐TESPMA‐Gel‐CaP, Ti‐TESPMA‐Gel, Ti‐TESPMA, and Ti. E) HR‐TEM images and SAED graphs of the gelatin‐CaP composite.

In this work, a feasible, simple, and low‐cost route has been established to fabricate Gel‐CaP coatings on a titanium surface to endow it with osteoconductive ability with titanium (**Figure** **1**). Briefly, titanium plates are cleaned by Piranha solution and pretreated with subsequent oxygen (O_2_) plasma to alter the surface chemistry terminated by hydroxyl groups, which is labeled as Ti–OH. Then the hydroxyl groups were chemically reacted with triethoxysilypropylmaleamic acid or 3‐(triethoxysilyl)propyl succinic anhydride yielding covalent silane attachment, which is called Ti‐TESPMA or Ti‐TPSA. Subsequently, gelatin was bound to the carboxyl groups via NHS/EDC activation chemistry labeled as Ti‐TESPMA‐Gel or Ti‐TPSA‐Gel. At last, these plates were immersed in a mineralizing solution to mimic the process of biomineralization for 7 days. The final product is called Ti‐TESPMA‐Gel‐CaP or Ti‐TPSA‐Gel‐CaP. The morphology of the titanium surface was analyzed by SEM after every procedure (**Figure** **2**A). The analysis revealed that Ti displays a porous surface after the surface treatments. The generation of pores is attributed to sub‐surface corrosion by piranha solution treatment. In comparison, the surfaces of the corresponding Ti‐TESPMA‐Gel or Ti‐TPSA‐Gel appear to be smoother after the gelatin graft. In addition, Gel‐CaP coatings were observed to have a porous, rough surface. Rough surfaces have been demonstrated to provide excellent mechanical properties for the interface and a three‐dimensional structure for cell adhesion.^[^ ^30^ ^]^ The insets in Figure 2A and Table S1, Supporting Information, show the EDS results of each compound. The EDS results demonstrated the appearance of silicon signals in Ti‐TESPMA or Ti‐TPSA and nitrogen signals in Ti‐TESPMA‐Gel or Ti‐TPSA‐Gel, confirming the success of the chemical coupling of the silanes resp. gelatin. The appearance of calcium and phosphorus in Ti‐TESPMA‐Gel‐CaP or Ti‐TPSA‐Gel‐CaP demonstrated a successful mineralization with calcium phosphate, but the Ca and P mass content was found to be low (Table S1, Supporting Information). Also, calcium phosphate did not form any aggregates on the surface, which indicates that it is incorporated within the gelatin layer. This finding is important since big calcium phosphate particles like HAP on the implant surface have the potential risk of being detached and can lead to inflammation.^[^ ^31^ ^]^ Moreover, the low solubility product of HAP is considered the bottleneck, and it is reported that HAP cannot dissolve even after nine month's implantation.^[^ ^32^ ^]^ On the contrary, calcium ions could easily be released from our titanium surface and then participate in the entire life cycle of bone formation.^[^ ^33^ ^]^

In order to further explore the chemical composition of the coatings, XPS measurements were performed. Figure 2B and Figure S1, Supporting Information, show the XPS survey spectra of the different compounds. The surface of Ti‐TESPMA‐Gel‐CaP and Ti‐TPSA‐Gel‐CaP showed the presence of elements of Ti, O, C, N, Si, Ca, and P, which are in accordance with EDS mapping results. As expected, Ca and P cannot be observed on Ti‐TESPMA‐Gel or Ti‐TSPA‐Gel. Si2p peaks could be observed at the binding energy of 101.58 eV, indicating Si–O bonds on the surface. Figure S2, Supporting Information, depicts the high‐resolution XPS spectra of the elements of oxygen (O 1s), calcium (Ca 2p), and phosphorous (P 2p) in the corresponding CaP coating compound. As for Ti‐TESPMA‐Gel‐CaP, the binding energy of O 1s is located at 531.2 eV, 531.8 eV, and 532.1 eV, which could be attributed to Ti–O bonded on the surface. P2p peaks can be observed at 132.8 eV, which can be deconvoluted into two separate peaks: 133.3 eV and 132.5 eV, indicating the existence of HPO_4_^2−^ and PO_4_^3−^. Furthermore, Ca 2p peaks can be observed at 351 eV and 347.4 eV, which can also be associated with calcium present in the Ca_3_(PO_4_)_2_. As for Ti‐TPSA‐Gel‐CaP, O 1s peaks located at 530.2 eV, and the measured P 2p showed the peak at 132.8 eV, which could be attributed to Ti–O and P–O bonds. The high‐resolution XPS spectrum of Ca 2p showed two peaks of Ca 2p_1/2_ (349.8 eV), and Ca 2p_3/2_ (346.1 eV), which could be assigned to bivalent calcium.^[^ ^34^ ^]^ The values measured here all represent the composition of the first few nanometers of the coating on the surface and might also reflect the situation deeper in the coatings. The detailed surface elemental composition is shown in Table S2, Supporting Information. The content of different elements (at.%) was determined by area under the curve fittings. The data clearly demonstrated that the atomic ratios of Ca/P in Ti‐TESPMA‐Gel‐CaP and Ti‐TSPA‐Gel‐CaP are 1.48 and 1.65 respectively. According to previous research, calcium‐deficient hydroxyapatite (CDHA) has Ca/P ratios ranging from 1.5 to 1.667, and amorphous calcium phosphate (ACP)’s ratio is within the scope of 1.2–2.2, which fits the compound of calcium phosphate on the surface.^[^ ^35^ ^]^ In conclusion, the coatings consisted of calcium phosphate.

### 2.2 Phase Determination of the Mineral Component of the Coatings

FTIR was first used to investigate the calcium phosphate on the surface of the different compounds (Figure 2C, Figure S3B, Supporting Information). Ti‐TESPMA or Ti‐TPSA each show a signal in the range between 1005 and 894 cm^−1^, which can be attributed to the Si–O group, while the sharp signals at 1704 cm^−1^ and the broad signals ≈1500–1700 cm^−1^ can be assigned to the carboxyl group.

Also, the amount of bound carboxy groups on the Ti‐TESPMA can be estimated by titration with toluidine blue O (TBO), which is known to quantitatively bind to carboxyl groups (Figure S4, Supporting Information). First, we used titania plates with six different concentrations of silane coupling moieties and made a calibration between TBO concentration and absorption at 630 nm. Then, the concentration of the carboxy groups from the grafted silane coupling agent of each sample on the modified Ti surface was determined via the bound TBO after the TBO had been released by acetic acid. With increasing concentration of the silane coupler on the titanium surface, the concentration of the surface grafted carboxyl group initially increased (Figure S5, Supporting Information). At a certain point, the concentration of COOH groups reached a maximum of 5.73 µmol cm^−2^ (6 wt.% silane coupling agent). This corresponds to a maximum of 35 COOH groups per nm^2^ on the Ti‐TESPMA surface theoretically. However, the concentration of bound carboxy groups slightly decreased again at a higher coupling agent concentration. These results showed that the bound silane on the surface reaches a maximum concentration bound to the surface despite the increasing TESPMA concentration. These results indicate that the silane grafting reaction was successful.

Meanwhile, the signal at 1563 cm^−1^ in Ti‐TESPMA‐Gel and Ti‐TPSA‐Gel suggested the presence of secondary amide, showing that the gelatin molecule was connected to the titanium substrate via a covalent amide bond with the silane coupling agents (triethoxysilypropylmaleamic acid and (triethoxysilyl)propyl succinic anhydride). As for the mineralized coatings, signals at 1015 cm^−1^ and 947 cm^−1^ were observed, which were attributed to the P–O stretching band.^[^ ^36^ ^]^ Nancy et al. also observed that the second derivative of the *v_3_* PO_4_ band changed from 992 to 1015 cm^−1^ during the process of ACP in the developing matrix preceding the formation of apatite.^[^ ^37^ ^]^ Overall, these results indicate that the phase of the mineral component of the coating may be ACP or poorly crystallized HAP.

In addition, giXRD was applied to gain a more detailed insight into the phase composition of the CaP coatings. Figure 2D shows XRD micrographs of the different compounds. Signals at 35, 38.4, 40.2, 52.9, and 62.9° were measured for every compound. These signals correspond to the (100), (002), (101), (111), and (002) planes of Ti, respectively. The XRD patterns of Ti‐TESPMA‐Gel and Ti‐TESPMA‐Gel‐CaP show similar Ti signals. The lack of additional reflexes indicates that the CaP on the surface is either amorphous or the CaP amount in the surface layer is too low to be detectable. All XRD patterns in Ti‐TPSA compounds (Figure S3A, Supporting Information) were similar to those in the Ti‐TESPMA compounds. That means CaP on the surface of Ti‐TPSA‐Gel‐CaP could also be inferred as amorphous calcium phosphate rather than poorly crystallized hydroxyapatite or its amount was too low for detection.

To further study the mechanism to underline the combination of calcium phosphate and gelatin, a 1 wt.% gelatin solution was selected to connect with CaCl_2_ and Na_2_HPO_4_ under similar mineralization conditions to those for the surface‐bound gelatin layer. TEM analysis showed that CaP‐gelatin composites exerted nanoparticle clusters with a hollow structure. Selected area diffraction (SAED) confirmed that the CaP‐gelatin compound was in amorphous form (Figure 2E). Furthermore, EDS in Figure S6, Supporting Information, clearly shows the elemental composition of the nanoparticles. Strictly speaking, the nucleation of free calcium phosphate‐gelatin composites and the nucleation of calcium phosphate on surface‐bound gelatin does not work in a similar way. First, some important functional groups of the surface‐bound gelatin are not available during nucleation in comparison to free gelatin. Furthermore, gelatin does not have the completely same structural features as functional collagen, the organic matrix in bone. However, gelatin as a denatured form of collagen, still retains some of the functional properties, enabling a relative comparison of the mineralization process. Some researchers suggested that pAsp could inhibit calcium phosphate nucleation in synergy with collagen fibers to control mineralization.^[^ ^21^, ^38^ ^]^ In that experimental setup, ACP could actively penetrate into the discrete spaces of collagen fibrils and grow in a specific crystalline orientation under the interaction of amino acids.^[^ ^21^ ^]^ Hence, it appears to be plausible that calcium phosphate on the coating of Ti‐TESPMA‐Gel‐CaP or Ti‐TPSA‐Gel‐CaP may be in the amorphous form.

### 2.3 Thickness, Surface Roughness, Wettability, and Mechanical Properties of the Surface Layers

**Figure 3:**
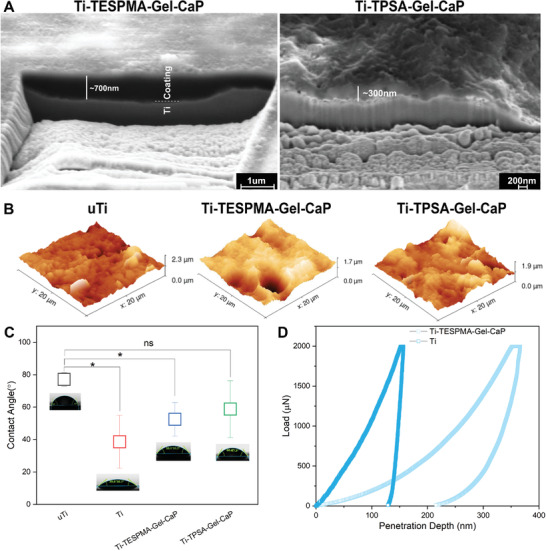
A) FIB‐SEM images of Ti‐TESPMA‐Gel‐CaP and Ti‐TPSA‐Gel‐CaP. Scale bars: 1 µm (left) and 200 nm (right). B) 3D AFM images of untreated Ti (uTi), Ti‐TESPMA‐Gel‐CaP, and Ti‐TPSA‐Gel‐CaP. C) Water contact angle for uTi, Ti, Ti‐TESPMA‐Gel‐CaP, and Ti‐TPSA‐Gel‐CaP. D) Load‐penetration depth curves of nanoindentation test for Ti and dry Ti‐TESPMA‐Gel‐CaP.

To determine the thickness of the covalently attached surface layer, it was sputtered with gold first and then observed by FIB‐SEM. **Figure** **3**A shows the SEM images of the FIB‐cuts, where the approximate thickness in Ti‐TESPMA‐Gel‐CaP was ≈600–800 nm while that in Ti‐TPSA‐Gel‐CaP was ≈200–400 nm. The variant thickness may attribute to different concentrations of silane coupling agents and different methods for grafting silane coupling agents.

In order to measure the approximate amount of gelatin of Ti‐TESPMA‐Gel, we chose Fluoresceineisothiocyanate (FITC) to attach to the amine groups of Ti‐TESPMA‐Gel and then measured the fluorescence intensity.^[^ ^39^ ^]^ Briefly speaking, different concentrations of gelatin solutions were utilized to react with EDC‐NHS‐activated Ti‐TESPMA. A calibration was made between different concentrations of FITC solution and absorbance. Thus, bound gelatin concentrations were obtained by calculating the corresponding absorbance for the unbound FITC left in solution. The results showed that the thickness of Ti‐TESPMA‐Gel was not related to the concentration of gelatin solutions before binding (Table S3, Supporting Information). The thickness of the gelatin coatings can be calculated from the amount of bound gelatin considering that the Ti‐TESPMA‐Gel coatings have approximately a thickness of 500–1000 nm depending on the concentration of the gelatin solution in the reaction (Table S3, Supporting Information). For 1% of gelatin solution, applied in the reaction to prepare Ti‐TESPMA‐Gel‐CaP, the thickness of the swollen gelatin layer is 680 nm. This confirms the experimental result from FIB‐SEM, which showed that the thickness of the dry Ti‐TESPMA‐Gel‐CaP layer was in the range of 700—800 nm. Nanometric apatite has been confirmed to offer calcium and orthophosphate ions for the bone “remodeling” process^[^ ^35^ ^]^ and our FiB‐SEM shows that the coatings have a sub‐micron‐scale thickness. The unaggregated nano‐ACP in the gelatin layer could facilitate the new bone formation at the interface of bone and implant.

Furthermore, high‐resolution AFM images revealed the roughness of the CaP coatings. In Figure 3B, the roughness with CaP coatings does not seem to change dramatically compared with untreated Ti (Ti‐TESPMA‐Gel‐CaP, 188 ± 91 nm; Ti‐TPSA‐Gel‐CaP, 171 ± 40 nm compared to Ti, 154 ± 42 nm). It was reported that a micron‐nanoscale modification of an implant surface would be advantageous for the binding of a variety of proteins, which can combine with selective receptors of osteoblasts to influence osteoblast proliferation and maturation, eventually leading to new bone formation.^[^ ^30^ ^]^

In terms of altering the interaction of the cell with the surface, surface energy or wettability is reported to be an important factor.^[^ ^40^ ^]^ A static water contact angle (WCA) was measured from two Gel‐CaP coatings and control samples. As shown in Figure 3C, the WCA of untreated Ti was nearly 77.06^o^ ± 4.26^o^. The WCA of Ti decreased significantly due to the piranha solution treatment. After grafting coatings, the mean WCA in Ti‐TESPMA‐Gel‐CaP and Ti‐TPSA‐Gel‐CaP were 52.47^o^ ± 10.36^o^ and 58.72^o^ ± 17.58 ^o^ respectively. This indicates that the surface of the CaP coatings is more hydrophilic and therefore more suitable for osseointegration by attracting more proteins to promote the adhesion of osteoblasts.^[^ ^40^, ^41^ ^]^

The mechanical properties of Ti‐TESPMA‐Gel‐CaP were investigated by nanoindentation in the dry state (Figure 3D). The values of nanohardness and Young's modulus were calculated and are displayed in Table S4, Supporting Information. Ti‐TESPMA‐Gel‐CaP showed a drop in elastic modulus compared to Ti (i.e., Ti‐TESPMA‐Gel‐CaP 18.20 ± 1.78 GPa and Ti 125.15 ± 15.56 GPa), which is much closer to bone's elastic modulus (7 – 30 GPa).^[^ ^42^ ^]^ Stress shielding is a major problem for titanium implants, and it would easily lead to new bone injuries.^[^ ^43^ ^]^ In this line, the Gel‐CaP coatings have desirable mechanical properties for osseointegration. Moreover, the ratio of nanohardness (H) and Young's modulus (elastic modulus, E) was calculated to evaluate the elastic strain to failure of the coating.^[^ ^44^ ^]^ The results showed a higher H/E ratio of Ti‐TESPMA‐Gel‐CaP than Ti (0.034 compared to 0.022, respectively), which may indicate better wear resistance. Some researchers proposed that HAP or poorly crystalline HAP is always considered responsible for brittleness in a load‐bearing application.^[^ ^45^ ^]^ In this regard, our CaP coatings’ mechanical property was improved by the addition of gelatin. From this viewpoint, surface modification of titanium with covalently bound Gel‐CaP could be a promising addition to titanium implants.

### 2.4 Release of Calcium Ions from the Coatings

**Figure 4:**
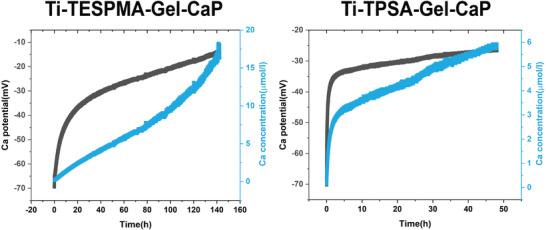
Calcium release curve from measuring Ca potential by Titration on Ti‐TESPMA‐Gel‐CaP and Ti‐TPSA‐Gel‐CaP.

Despite a solubility product of only 10^−25^, amorphous calcium phosphate was reported^[^ ^46^ ^]^ to release calcium ions in water or body fluid to bind to acidic proteins and create supersaturation conditions of surrounding biological fluids for bone mineral nucleation,^[^ ^35^, ^47^ ^]^ since the solubility product of biological apatite is much lower with 10^−50^ for calcium deficient carbonated HAP.^[^ ^46^ ^]^ Therefore, the calcium potential was measured in solution to estimate the amount of calcium release from the Gel‐CaP surface layers. In **Figure** **4**, it is displayed that calcium ions were rapidly released from the surface of Gel‐CaP. The calcium concentration reached 3.37 µmol l^−1^ and 5.56 µmol l^−1^, respectively, after 12 h in Ti‐TPSA‐Gel‐CaP and after 48 h in Ti‐TESPMA‐Gel‐CaP. Later, the Ca concentration in Ti‐TESPMA‐Gel‐CaP arrives at nearly 16 µmol l^−1^ after 140 h, and that in Ti‐TPSA‐Gel‐CaP can reach 5.5 µmol l^−1^ after 45 h. This is interesting to note since the Gel‐CaP Surface layers should, in principle, be the same, but they exhibit a difference in Ca^2+^ release after 45 h. This can be explained by the 1/3 lower surface Ca^2+^ concentration in Ti‐TPSA‐Gel‐CaP as compared to Ti‐TESPMA‐Gel‐CaP (Table S2, Supporting Information). Also, from XPS and EDS, the Ca^2+^ concentrations in the entire Ti‐TPSA‐Gel‐CaP layer are considerably lower than in Ti‐TESPMA‐Gel‐CaP (Tables S1 and S2, Supporting Information), which could explain the higher Ca^2+^ release from in Ti‐TESPMA‐Gel‐CaP. Nevertheless, it needs to be considered that in both cases Ca^2+^ was still released after the end of the measurement with an even increasing release rate after 100 h for Ti‐TESPMA‐Gel‐CaP. Thus, it can be inferred that both surface layers release Ca^2+^ even after days, which is advantageous for new bone generation. Interestingly, Xue et al. found that the expression of BSP, a later‐phase marker of osteogenic differentiation, was higher in low concentrations of Ca^2+^ media than in high concentrations of Ca^2+^ media. Besides, in vivo results showed that the higher Ca^2+^ content delays the maturation of cells and allows greater proliferation before reaching maturation, leading to increased bone volume while reaching the same terminal fate.^[^ ^48^ ^]^ The results were similar to our subsequent biological experiments. MC3T3 cells cocultured in Ti‐TESPMA‐Gel‐CaP showed better adhesion than in Ti‐TPSA‐Gel‐CaP, while the osteogenic‐related markers like BSP expressed higher in Ti‐TPSA‐Gel‐CaP than that in TESPMA‐Gel‐CaP. In this regard, releasing Ca ions is suitable and imperative to maintain the extracellular concentration of calcium to promote bone formation.^[^ ^49^ ^]^

### 2.5 Biocompatibility of the Modified Surfaces

**Figure 5:**
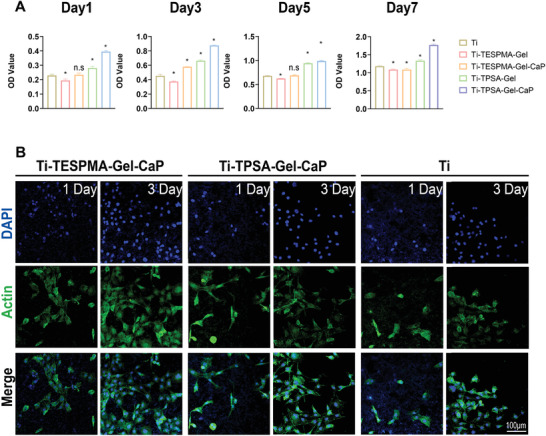
A) Results of Cell Counting Kit‐8 (CCK 8) test for MC3T3 cells cultured on Ti, Ti‐TESPMA‐Gel, Ti‐TESPMA‐Gel‐CaP, Ti‐TPSA‐Gel, and Ti‐TPSA‐Gel‐CaP for 1 day, 3 days, 5 days, 7 days. *p < 0.05. B) Immunofluorescence images of MC3T3 cells cultured on Ti, Ti‐TESPMA‐Gel‐CaP, and Ti‐TPSA‐Gel‐CaP for one day, three days after seeding. The cell nuclei exhibit blue signal stained with DAPI, and the cytoskeleton shows green fluorescence stained with FITC‐phalloidin. (The scale bar can be applied to every picture.)

In this study, Gel‐CaP surface layers on titanium implants with submicron thickness were designed in order to increase osseointegration. Thus, cell responses or cellular behavior to Gel‐CaP surface layers were investigated in this study. At first, the viability of MC3T3 osteoblast cells cultured on Ti, Ti‐TESPMA‐Gel, Ti‐TPSA‐Gel, Ti‐TESPMA‐Gel‐CaP, and Ti‐TPSA‐Gel‐CaP was quantified by Cell counting Kit‐8 (CCK8) (**Figure** **5**A). The amounts of cells in the five groups showed an upward trend for seven days. After one day, Ti‐TESPMA‐Gel‐CaP did not experience an apparent cell growth compared to Ti. However, compared with Ti, Ti‐TPSA‐Gel‐CaP, or Ti‐TESPMA‐Gel‐CaP, it exhibited a higher OD value of cell density from 3 days to 7 days after cell seeding. Concurrently, all Gel‐CaP coatings showed a higher OD value than corresponding gelatin coatings at each observation time.

**Figure 6:**
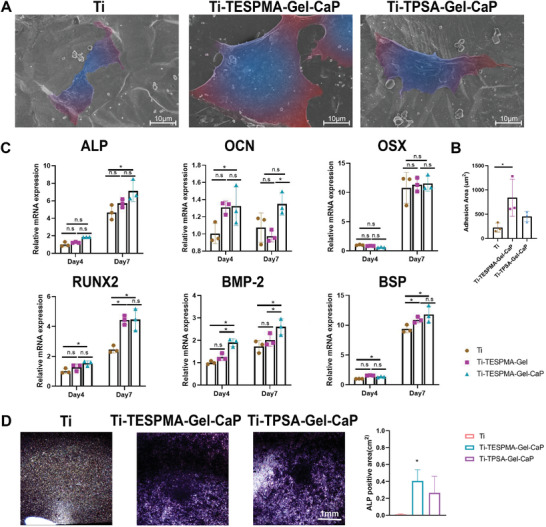
A) SEM images of MC3T3 cells seeded on Ti, Ti‐TESPMA‐Gel‐CaP, and Ti‐TPSA‐Gel‐CaP for 24 h. B) statistical results of the adhesive area proportion of MC3T3 cells on different groups. C) Real‐time PCR analysis of osteogenic differentiation genes of MC3T3 after culturing for 4 and 7 days on the three groups. ALP, RUNX2: early‐stage markers of osteogenic differentiation, OSX, BMP‐2: middle‐stage markers of osteogenic differentiation, OCN, BSP: later‐stage markers of osteogenic differentiation. D) ALP staining images of MC3T3 cultured on Ti, Ti‐TESPMA‐Gel‐CaP, and Ti‐TPSA‐Gel‐CaP for 7 days. (*p < 0.05).

Moreover, the morphology and adhesion of MC3T3 cells were shown by immunofluorescence (IF) staining and SEM. In Figure 5B, all MC3T3 cells showed good adhesion on Ti, Ti‐TESPMA‐Gel‐CaP, and Ti‐TPSA‐Gel‐CaP. However, the MC3T3 cells that adhered to Gel‐CaP coatings exerted much more filopodia and lamellipodia than those on the Ti surface, which indicated better attachment. The amount of MC3T3 cells was significantly higher on Gel‐CaP coatings than the Ti group after three days of cell spreading. **Figure** **6**A,B shows the SEM images of MC3T3 cells cultured with Ti, Ti‐TPSA‐Gel‐CaP, and Ti‐TESPMA‐Gel‐CaP one day after cell seeding. The adhesive area proportion on Ti‐TESPMA‐Gel‐CaP was significantly higher than that on Ti. The SEM images exhibited similar results as the immunofluorescence (IF) results shown above. It is distinct that MC3T3 cells on Gel‐CaP coatings were more prolongated and spindle‐shaped than those on the Ti. Vinculin is a ubiquitously expressed actin‐binding protein and is used as a marker for cell‐extracellular matrix junctions.^[^ ^50^ ^]^ We also tested the expression of Vinculin on Ti, Ti‐TESPMA‐Gel, and Ti‐TESPMA‐Gel‐CaP. Gel‐CaP surfaces showed a higher expression of Vinculin than other surfaces (Figure S7, Supporting Information). These results further indicated that Gel‐CaP coatings have an excellent capacity for promoting initial cell adhesion and cell proliferation.

### 2.6 Osteogenic Ability of the Coatings

Furthermore, the osteogenic ability of the Gel‐CaP coatings was also studied by polymerase chain reaction (PCR) and alkaline phosphatase (ALP) staining. The osteogenic genes of ALP, Runt‐related transcription factor 2 (RUNX2), Osterix (OSX), Bone morphogenetic protein 2 (BMP2), bone sialoprotein (BSP) and Osteocalcin (OCN) were investigated by reverse transcription‐polymerase chain reaction (RT‐PCR) at day 4 and 7 (Primer sequences used are shown in Table S5). Gel‐CaP surfaces experienced a remarkably increased expression over time compared with Ti. Furthermore, the expression of RUNX2, ALP, OSX increased significantly in Ti‐TESPMA‐Gel‐CaP compared with Ti on day 4. On day 7, the expression of RUNX2, ALP, OSX, BSP, and BMP‐2 rose more in Ti‐TESPMA‐Gel‐CaP than in Ti (Figure 6C). Most genes expressed in Ti‐TPSA‐Gel‐CaP increased remarkably more than in Ti on days 4 and 7 (Figure S8, Supporting Information). Besides, BMSCs have been considered to be the progenitor cells for skeletal tissues.^[^ ^51^ ^]^ We utilized BMSCs to complement the conclusion that Gel‐CaP coatings could accelerate MSCs differentiation at the mRNA level. The results exerted that the expression of ALP, RUNX2, OCN, BMP‐2, BSP, and OSX increased significantly in the Ti‐TESPMA‐Gel‐CaP group rather than in the Ti group on day 7 (Figure S9, Supporting Information).

To some extent, ALP activity is the early marker of osteoblast differentiation since ALP can provide phosphate at the early stage of mineralization. RUNX2 is an osteoblast‐specific transcription factor that plays a central role in osteoblast differentiation, and the expression of RUNX2 could be upregulated in immature osteoblasts.^[^ ^52^ ^]^ OSX is a zinc‐finger‐containing transcription factor located downstream of RUNX2, responsible for osteoblast differentiation and bone mineralization.^[^ ^53^ ^]^ BMP‐2 occupies an essential position in stimulating the differentiation of mesenchymal cells into osteoblasts and could be detected during the whole stage of osteoblast differentiation.^[^ ^54^ ^]^ BSP, as a matricellular protein, can further increase the hydroxyapatite nucleation during bone mineralization and can be regarded as a marker in the later stage of osteogenesis. OCN can also be thought of as a final‐stage osteoblastic differentiation marker. Mahamid et al. found that ACP is a major component of the forming fin bones of zebrafish,^[^ ^17^ ^]^ and Lotsari demonstrated a detailed transformation mechanism of ACP to apatite platelet‐like crystals.^[^ ^19^ ^]^ Besides, several hypotheses were proposed to explain the mechanism of how ACP infiltrates into collagen fibrils, such as electrostatic interaction^[^ ^21^ ^]^ and the balance between electroneutrality and osmotic equilibrium.^[^ ^38^ ^]^ Accumulating evidence indicates a transformation from ACP to HAP in bone's nucleation and growth of mineral crystals. Our results showed that Gel‐ACP coatings could release Ca ions, which means ACP is more soluble and flexible for reorganization and fusion. Moreover, ALP, BSP, and OCN showed higher expression on Gel‐ACP coatings than on Ti. It may attribute to the efficiency in mineralization and convenience in delivery of ACP. All that means the Gel‐CaP coatings can improve the osteogenic ability of osteoblasts throughout the entire lifecycle of cells.^[^ ^55^ ^]^

ALP staining also showed the difference between Gel‐CaP coatings and Ti directly. Figure 6D demonstrated that Gel‐CaP coatings exerted a deeper color and larger colored area than Ti. Figure S10, Supporting Information, shows the upregulation of RUNX2 and OSX by Gel‐CaP coatings. These results are analogous to that in PCR. To conclude, our Gel‐CaP coatings could greatly impact the osteogenic activity of osteoblasts through the nanometric CaP, surface wettability, roughness, and the release of calcium ions.

## 3 Conclusions

This work demonstrated a facile method to fabricate Gel‐CaP coatings on titanium implants to mimic the initial bone biological apatite. We applied a literature‐reported strategy^[^ ^29^ ^]^ for silane‐mediated collagen coupling to titanium surfaces to gelatin and mineralized the covalently bound surface layers with submicron thickness with ACP, which is an advantageous precursor for remodeling to bone due to its much higher solubility (factor 10^25^ in the solubility product) as compared to HAP.

This should lead to rapid bone formation at the interface of bone and implant. To the author's knowledge, it is the first time to fabricate a Gel‐ACP composite coating successfully on titanium implants. The amorphous phases in the coatings were found to facilitate calcium release by titration methods. The excellent MC3T3 cell viability and higher expression of osteogenic genes in osteoblasts demonstrated the “bio‐active” response on the interface, which can be attributed to calcium release. In addition, this coating proved to exert advantageous nano‐topography,  good surface roughness, and high wettability, further promoting cell adhesion. On the other hand, the mechanical properties of the surface layer are rather close to those of natural bone so that the covalently bound surface layer might prevent “stress shielding”, which is observed for conventional titanium implants. Therefore, the reported covalently bound Gel‐CaP surface layer is a significant improvement for commonly used titanium implants with much better bioactivity towards osseointegration.

## 4 Experimental Section

### Preparation of Substrates

Commercial pure titanium discs (10 × 10 × 2 mm, L × W × H) were used as substrates. Titanium discs were first ground and polished with grinding paper from 800 to 4000 grids and then ultrasonically cleaned in acetone, ethanol, and distilled water. After drying with nitrogen, titanium discs, and silicon wafers were immersed in a 2:1 (v/v) mixture of concentrated H_2_SO_4_ (Sigma–Aldrich, Germany) and 30% H_2_O_2_(Sigma‐Aldrich, Germany) piranha solution at 25 °C for 1 h. The samples were then rinsed with distilled water three times and dried in N_2_ atmosphere at room temperature. The samples were labeled as Ti.

### Biomimetic Surface Preparation

In this study, triethoxysilypropylmaleamic acid (TESPMA; Gelest, Germany) and 3‐(triethoxysilyl)propyl succinic anhydride (TPSA; Sigma‐Aldrich, Germany) were grafted onto the titanium oxide or silicon wafer surface in different ways. The silane solution was prepared with 0.1 g TESPMA in 10 mL of absolute ethanol (TCI, German) containing 1% acetic acid. Then, titanium plates were soaked for 1 h at room temperature. Later samples were heated at 120 ^o^C for 2 h and labeled as Ti‐TESPMA.

As for Ti‐TPSA, surface silanization was carried out using the vapor method. The Ti, 1,1‐diiso‐propylethylamine (20 µL), and TPSA (60 ul) were placed inside a desiccator and left for 2 h under vacuum conditions. Then, samples were heated in an oven at 120 °C for 2 h. Afterward, they were immersed with 93.75 wt.% ethanol aqueous solution followed by heat treatment applying 90 °C for 1 hour (labeled as Ti‐TPSA).

Subsequently, Ti‐TESPMA or Ti‐TPSA was immersed into 70 mM EDC and 28 mM NHS solution in 50 mM MES buffer (pH = 5.0) at room temperature for 2 h according to literature.^[^ ^56^ ^]^ After the titanium samples were rinsed with distilled water thrice, 10 mM HEPES buffer containing 1 wt.% gelatin (pH = 7.4) was used to soak titanium samples at room temperature for 1 h. Afterward, the samples were washed with distilled water and dried under N_2_ flow (labeled as Ti‐TESPMA‐Gel or Ti‐TPSA‐Gel, respectively). For each titanium sample, a CaCl_2_ (1.35 mM in water) solution in HEPES buffer was prepared. The Ti‐TESPMA‐Gel or Ti‐TPSA‐Gel was immersed into the solution followed by the addition of pAsp (10 µg mL^−1^ in water) and Na_2_HPO_4_ (1.35 mM in water) via a peristaltic pump (3 mL min^−1^). All procedures were maintained at a pH level of 9 and finished after ≈15 min. Finally, the sample solution was placed inside an oven which was set to 37°C for 7 days. The samples were labeled as Ti‐TESPMA‐Gel‐CaP or Ti‐TPSA‐Gel‐CaP.

### Surface Characterization of Gel‐CaP Coatings

The morphology of coatings was observed by scanning electron microscopy (SEM; Zeiss Gemini500, Germany), and energy dispersive X‐ray spectroscopy (EDS) was performed with an Oxford instruments X‐max detector. The X‐ray photoelectron spectroscopy (XPS) spectra were obtained with a PHI Quantera SXM equipped with an aluminum anode (15 kV, 1486.6 eV) and a quartz monochromator to analyze the surface composition and chemical state. During the process, the pressure was kept below 2 × 10^−7^ Pa. Spectra analysis was performed with CasaXPS software, which includes a Shirley background subtraction and peak separation adopting mixed Gaussian−Lorentzian functions in a least‐squares curve fitting program. Contact angle measurements were performed by a surface contact angle instrument (DSA25, Krüss, Germany). Three different samples were analyzed (two water drops/sample). Each sample was taken for ten measurement points and the reported WCA is the average of all values obtained. The surface hardness was measured by a nanomechanical test instrument (Hysitron Ti980 Triboindentor, Germany) with a 2N load on six random locations of the sample surface, and the average value was calculated. Fourier transform infrared (FT‐IR) spectroscopy (Perkin Elmer Spectrum 100, Germany) was applied to study the vibrational modes of coatings in the infrared region within the region of 4000–550 cm^−1^. The phase composition was recorded by X‐ray diffraction (XRD; Bruker D8 Discover, Germany). Data were collected at room temperature over a 2*θ* range of 0–65^o^ with an incident degree of 1.5^o^ and a step time of 10 800 s. For STEM‐EDS analysis, 10 mM HEPES solution containing 1 wt% gelatin (PH = 7.4) was prepared. CaCl_2_ (1.35 mM in water) and pAsp (10 ug/mL in water) were added into the gelatin solution followed by the slow addition of Na_2_HPO_4_ (1.35 mM in water) while maintaining a pH value of 9. Stirring at 37 °C was maintained for 7 days. Afterward, the solution was diluted 100 times. 100 µL of the suspension was drop‐casted onto a carbon‐coated copper grid and dried in air for high‐resolution transmission electron microscopy characterization (TEM; JEM‐2200FS, JEOL, Japan). The morphologies of the gelatin‐CaP compound were examined using Scanning transmission electron microscopy (STEM, JEOL‐2200FS, Japan) at an accelerating voltage of 200 kV. A TEM‐energy dispersive X‐ray analysis (TEM‐EDX) was performed to measure the calcium (Ca) and phosphorus (P) contents of the CaP‐gelatin composite.

### Thickness or Toughness of Gel‐CaP Coatings

Atomic force microscopy (AFM; JPK, Japan) was applied to measure the three‐dimensional morphology and roughness of the Gel‐CaP coating. The thickness of Gel‐CaP coatings was examined by focused ion beam‐scanning electron microscopy (FIB‐SEM; Zeiss, Germany). The acceleration voltage and current were set to be 30 kV and 50 pA at a normal incident angle, respectively. Finally, the etching section was polished with 100 pA current to achieve a clearer cross‐section, and the thickness was measured.

### Quantification of Concentration of Surface‐Grafted Silane in Ti‐TESPMA

Toluidine blue O (TBO) staining was applied to determine the graft concentration of silane coupling agent. First, UV spectroscopy (Varian Cary 50 spectrometer, Germany) was introduced to measure the optical density of different known concentrations of TBO solutions at 630 nm, and a calibration curve was generated. Different samples immersed in several known concentrations of silane coupling agent were prepared. An aqueous solution of TBO (0.5 mM) was prepared and adjusted to a pH of 10 by adding 0.1 mM NaOH. Then, 1 mL TBO solution was added to each 1 cm^2^ Ti‐TESPMA. After 5 h, the COOH group from Ti‐TESPMA could coordinate with TBO at room temperature. Residual compounds were removed by rinsing with 0.1 mM NaOH solution. 1 mL of 50% (v/v) acetic acid was used to desorb the coordinated TBO from the surface. The amount of COOH groups was calculated from the optical density of the TBO dye at 630 nm.

### Quantification of the Theoretical Thickness of Ti‐TESPMA‐Gel

The optical density of various concentrations of fluorescein isothiocyanate (FITC) was first measured with UV spectroscopy (Varian Cary 50 spectrometer, Germany) at 490 nm to generate a calibration curve. Then, FITC was applied to bind to the amino groups of gelatin. Optical density was measured to obtain the difference before and after immersion of EDC‐NHS‐activated Ti‐TESPMA in different concentrations of FITC‐labeled gelatin (0.1%, 1%, 5%, 10% by weight). The bound amount of gelatin on the surface of Ti‐TESPMA‐Gel can be inferred subsequently, and the specific volume of gelatin could also be calculated by using Equation 1:

(1) $$V_{\mathrm{bound}\mathrm{gelatin}}=W_{\mathrm{bound}\mathrm{gelatin}}/\rho$$

where *ρ* is the relative density of gelatin (*ρ* = 1.35 g ml^−1^), *V* is the volume of bound gelatin, *W* is the weight of bound gelatin. The theoretical thickness of Ti‐TESPMA‐Gel can be calculated by Equation 2

(2) $$T_{\mathrm{bound}\mathrm{gelatin}}=V_{\mathrm{bound}\mathrm{gelatin}}/S_{\mathrm{bound}\mathrm{gelatin}}\times Q_{m}$$

where *T*_bound gelatin_ is the thickness of the specific volume of gelatin, *S*_bound gelatin_ is the surface area of the coating, and *Q*_m_ is the equilibrium mass swelling ratio, which was calculated using Equation 3

(3) $$Q_{m}=(m_{s}-m_{p)}/m_{p}\times 100\%$$

where *m_s_* and *m_p_* are swollen gelatin mass and unswollen gelatin mass.

### Release of Calcium Ions by Ti‐TESPMA‐Gel‐CaP or Ti‐TPSA‐Gel‐CaP

pH and calcium concentration were measured using a glass electrode (Metrohm Unitrode flat membrane 6.0256.100) with internal reference and a calcium‐selective electrode using polymer‐based ion‐selective electrodes (ISE, Metrohm 6.0508.110) respectively.

Calcium ISE electrode was calibrated by titration of calcium chloride 10 mM solution into 10 mL of ultrapure water set at a desired pH (previously adjusted by addition of NaOH) while a gentle stream of nitrogen was flushed over the calibration sample to limit CO_2_ uptake during measurement and exclude any unwanted calcium ion binding. Calcium was added via an automated titration setup (Metrohm 906 Titrando and a Metrohm 800 Dosino dosing units) operated via the software Tiamo 2.5.

pH‐electrode calibration was carried out by three‐point calibration with standard pH buffer solutions from Mettler‐Toledo with the product numbers: pH  =  4.01: 51 302 069; pH  =  7.00: 51 302 047; pH  =  9.21: 51 302 070.

Then, the titanium sample was placed into 10 mL of distilled water followed by monitoring the calcium potential for a certain time (2 days or 7 days).

### Cell Viability Test and Cell Adhesion Test

MC3T3‐E1 cell line was bought from ATCC (U.S.A). MC3T3 was seeded with Ti, Ti‐TESPMA‐Gel, Ti‐TESPMA‐Gel‐CaP, Ti‐TPSA‐Gel, Ti‐TPSA‐Gel‐CaP three groups at a density of 5000 per well. After 1 day, 3 days, 7 days, and 10 days of culture, cell viability was assayed using a Cell counting Kit‐8 (CCK8; Beyotime, China) and observed according to the manufacturer's instructions. As for SEM observation, 5000 MC3T3 cells were cocultured with different groups. After 24 h, cells were washed with PBS and fixed with 4% PFA for 30 min. Finally, samples were washed with *tert*‐butyl alcohol to replace the ethanol and were dried with a vacuum freeze‐dryer.

As for the adhesion test, 5000 MC3T3 cells were plated to Ti, Ti‐TESPMA‐Gel‐CaP respectively. After 1 day and 3 days of culture, cells were washed with PBS and fixed with 4% PFA for 30 min. They were then stained with Phalldin (Sigma) for 40 min and DAPI for 5 min at 37 °C. After washing with PBS, the cells and materials were observed under confocal laser scanning microscopy (Nikon Ni‐U/Fl/Ri2/Elements‐D, Nikon, Japan). The images were reconstructed by Imaris software.

### Osteogenic Cell Differentiation with Gel‐CaP Coating

MC3T3 cells were plated with Ti, Ti‐TESPMA‐Gel, Ti‐TESPMA‐Gel‐CaP at a density of 5000 per well. After 4 days and 7 days of culture, total RNA was extracted using the RNAiso Plus reagent (Takara Bio, Japan), reverse transcribed using a PrimeScript RT reagent kit with gDNA Eraser (Perfect Real Time) (Takara Bio), and then subjected to qPCR analysis using SYBR Mix and a LightCycler System (Roche, Switzerland) according to the manufacturer's protocol. Fold changes of mRNA were calculated by the 2−ΔΔ*C_t_* method after normalization to the expression of a housekeeping gene (GADPH).

MC3T3 cells were plated with Ti, Ti‐TESPMA‐Gel, Ti‐TESPMA‐Gel‐CaP at a density of 10 000 per well. After 7 days, Alkaline phosphatase (ALP) stain kit was applied to identify the activity of osteogenic cells. The 24‐well plates were fixed by PFA and cleaned by PBS. Incubating solution was added to plates for 60 min and then cleaned by PBS. The images were observed by microscopy(Olympus, Japan) and analyzed by Image J software.

### Statistical Analysis

The data is displayed as mean value and the corresponding standard deviation (SD). For statistical analysis, a one‐way ANOVA test was conducted, and values of *p* < 0.05 were deemed to be statistically significant.

## Conflict of Interest

The authors declare no conflict of interest.

## Supporting information

Supporting information

## Data Availability

The data that support the findings of this study are available from the corresponding author upon reasonable request.
