# Supplementary material for: A Bioinspired Gelatin–Amorphous Calcium Phosphate Coating on Titanium Implant for Bone Regeneration
Source: Adv Healthc Mater. 2023 Apr 2;12(20):2203411. doi: 10.1002/adhm.202203411 (PMC11468875; doi:10.1002/adhm.202203411)
Supplement: Supplementary file 1 — Supporting information [file ADHM-12-2203411-s001.pdf]

ADVANCED HEALTHCARE MATERIALS

Supporting Information

for *Adv. Healthcare Mater.*, DOI 10.1002/adhm.202203411

A Bioinspired Gelatin–Amorphous Calcium Phosphate Coating on Titanium Implant for Bone Regeneration

Yanhuizhi Feng, Di Wu, Jennifer Knaus, Sascha Keßler, Bing Ni, ZongKun Chen, Johnathan Avaro, Rui Xiong, Helmut Cölfen and Zuolin Wang**

Supporting information

A bioinspired gelatin–amorphous calcium phosphate coating on Titanium implant for bone regeneration

Yanhuizhi Feng, Di Wu, Jennifer Knaus, Sascha Keßler, Bing Ni, ZongKun Chen, Johnathan Avaro, Rui Xiong, Helmut Cölfen, and Zuolin Wang**

Table S1. Elemental composition of seven groups from EDS mapping.

samples	Ti(Wt.%)	O(Wt.%)	C(Wt.%)	Si(Wt.%)	N(Wt.%)	Ca(Wt.%)	P(Wt.%)
Ti-TESPMA-Gel-CaP	89.7	8.8	0.7	0.1	0.4	0.2	0.1
Ti-TESPMA-Gel	88.3	8.4	2.0	0.5	0.8		
Ti-TESPMA	81.1	12.9	3.4	2.1	0.6		
Ti	91.15	8.85					
Ti-TPSA-Gel-CaP	91.06	5.91	0.51	1.19	1.12	0.14	0.06
Ti-TPSA-Gel	94.2	4.3	0.1	0.2	1.2		
Ti-TPSA	85.1	3.5	11.3	0.2			

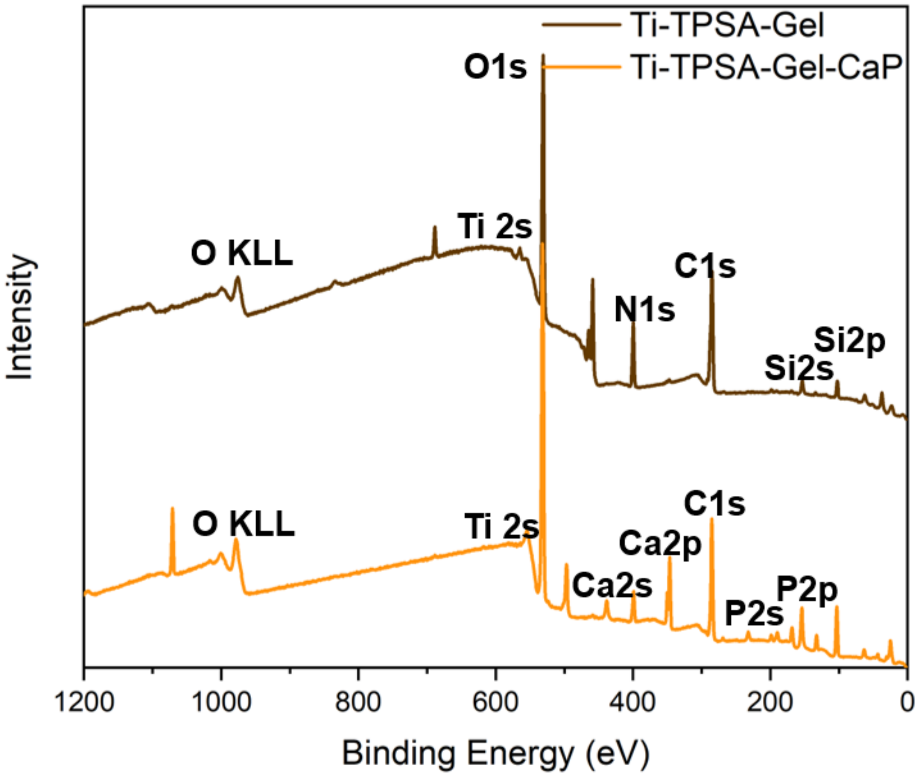

Figure S1. XPS survey spectra of Ti-TPSA-Gel and Ti-TPSA-Gel-CaP.

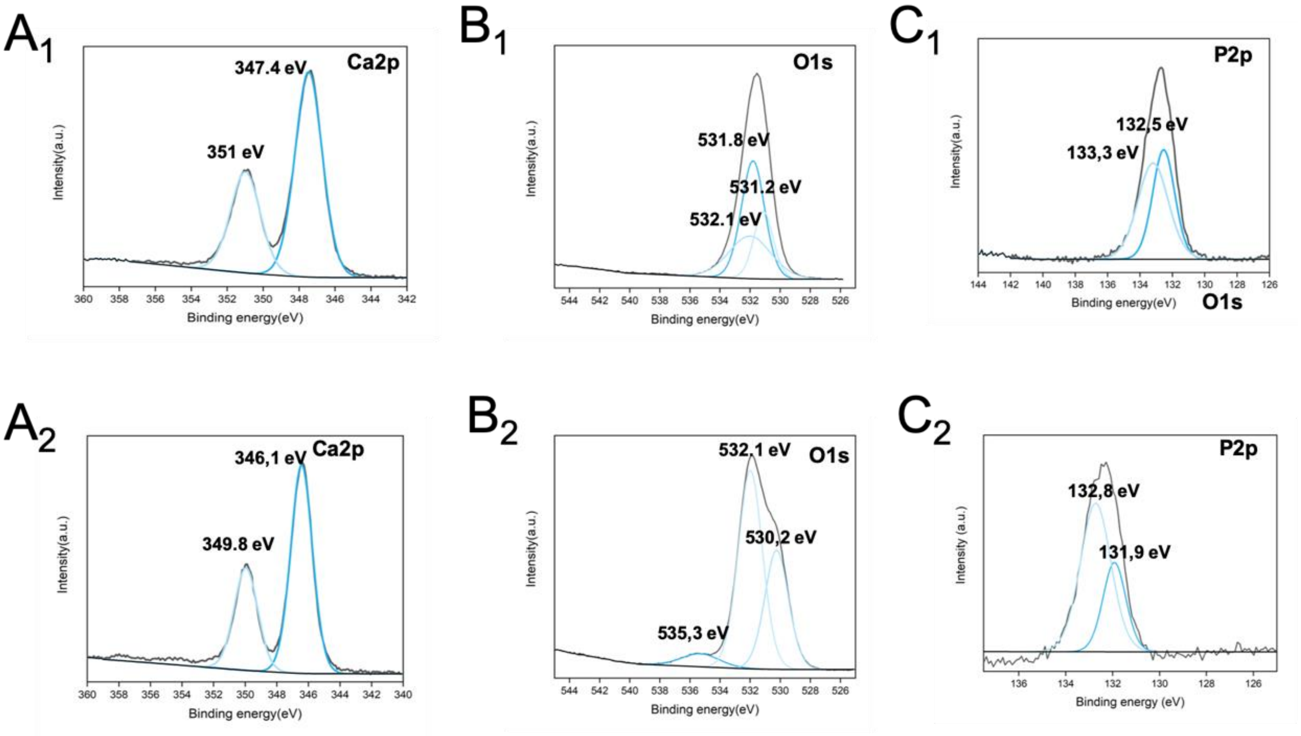

Figure S2. Highresolution XPS survey spectra of Ca 2p, O 1s, P 2p (A_1-C_1 correspond to Ti-TESPMA-Gel-CaP, A_2-C_2 correspond to Ti-TPSA-Gel-CaP).

Table S2. Elemental composition of the coatings from XPS.

samples	Ti(at.%)	O(at.%)	Ca(at.%)	P(at.%)	C(at.%)	N(at.%)	Si(at.%)
Ti-TESPMA-Gel-CaP	1.66	31.1	12.73	6.5	34.97	7.31	5.73
Ti-TESPMA-Gel		24.54			53.57	17.25	4.64
Ti-TPSA-Gel-CaP	0.56	38.78	8.43	3.91	24.71	4	19.61
Ti-TPSA-Gel	17.17	32.83			33.74	8.83	7.42

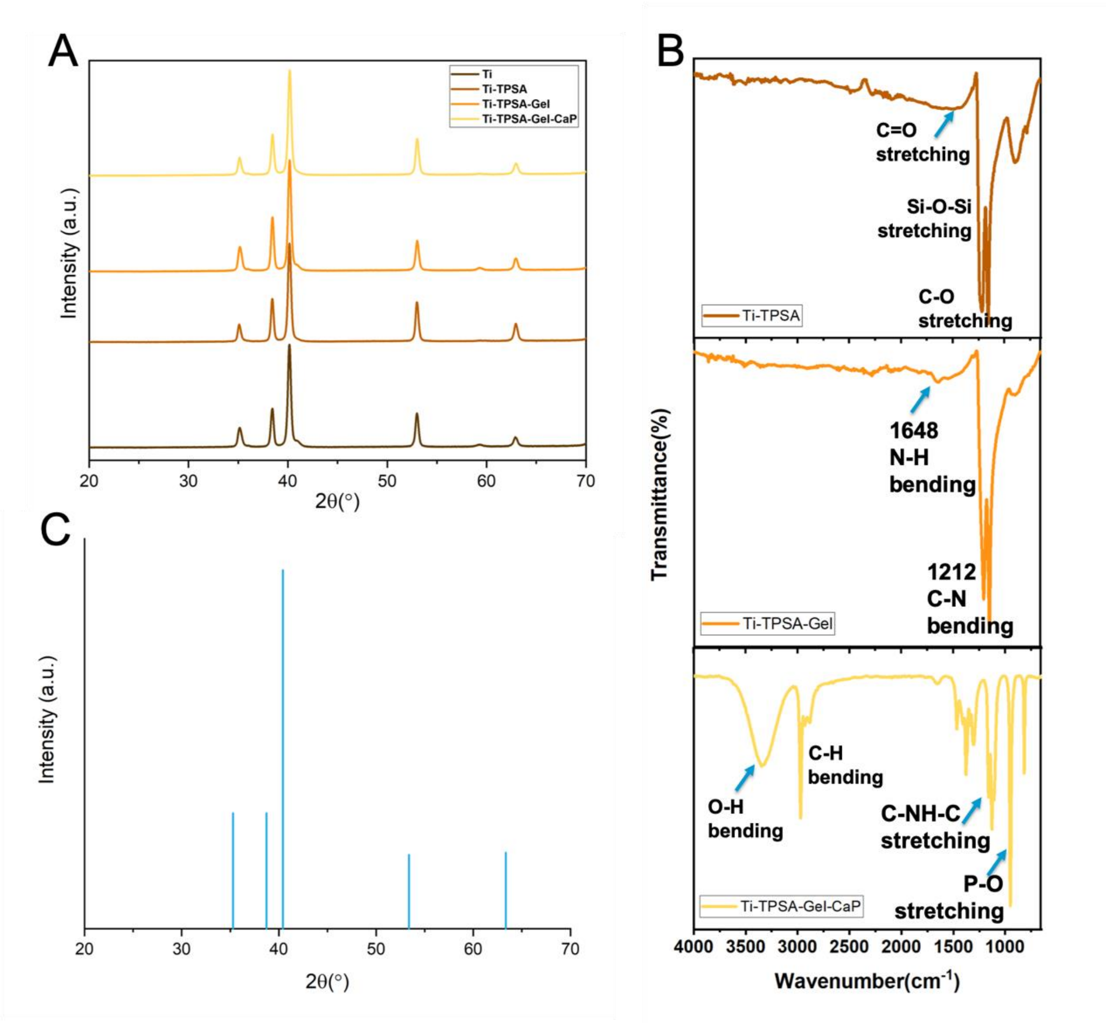

Figure S3. (A) XRD spectra of Ti-TPSA-Gel-CaP, Ti-TPSA-Gel, Ti-TPSA, Ti; (B) FT-IR spectra analysis of Ti-TPSA-Gel-CaP, Ti-TPSA-Gel, Ti-TPSA, Ti; (C) Assigned PXRD pattern of Titania (reference number: 96-901-2926).

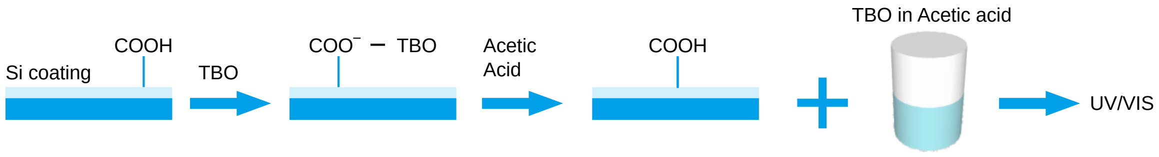

Figure S4. Schematic illustration for the determination of COOH concentration via the TBO method.

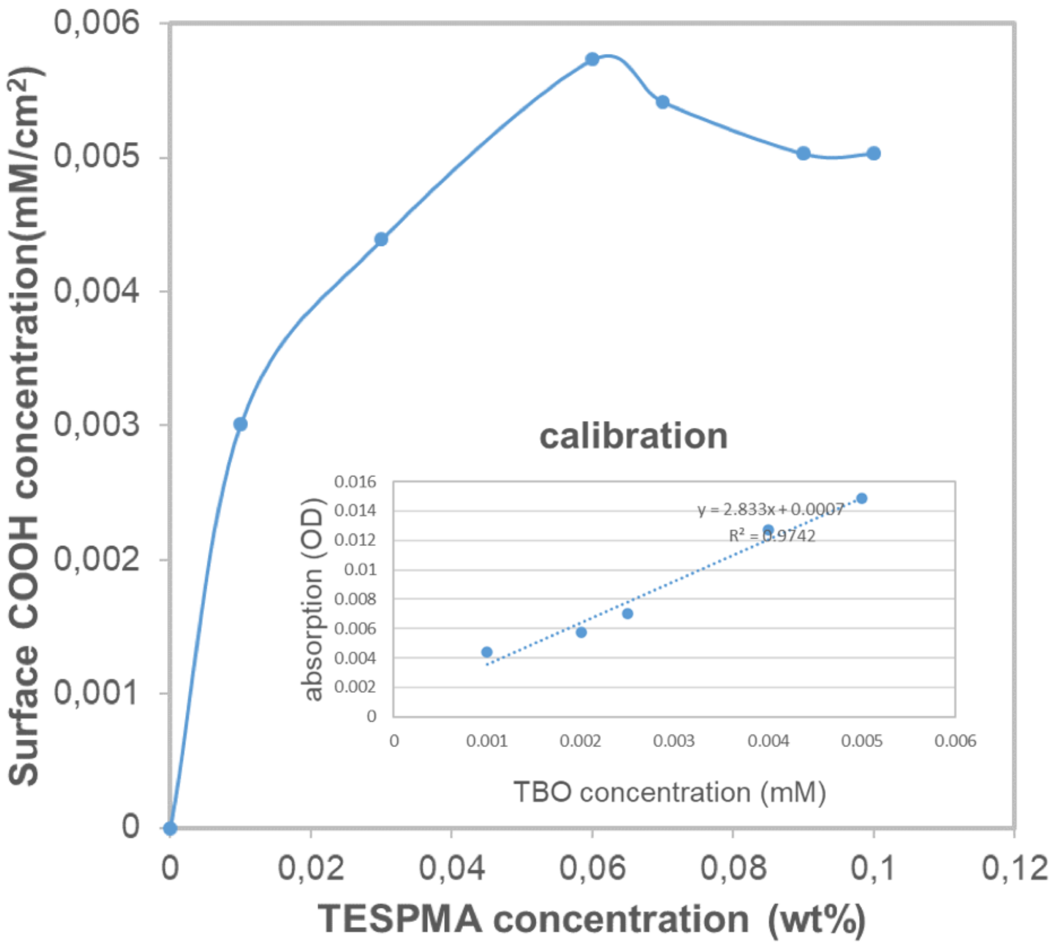

Figure S5. Line profile obtained from UV/VIS spectroscopy showing the effect of TESPMA concentration and ultimate grafted concentration of carboxyl groups on the titanium surface. Inset: The linear relationship of TBO concentration and Absorption at 630 nm measured by UV-Vis spectroscopy.

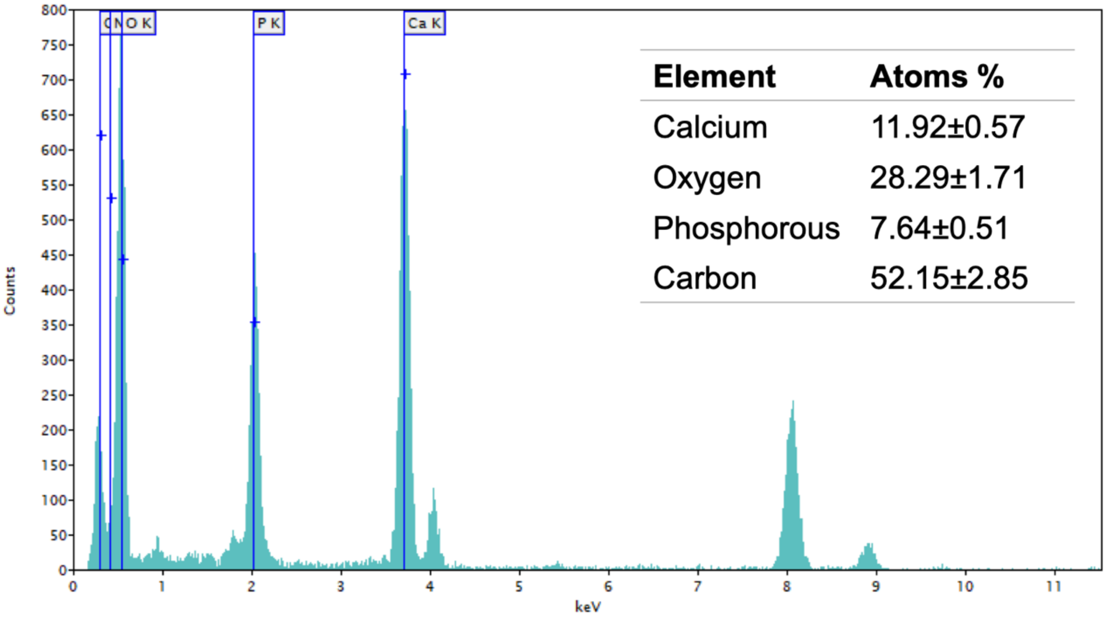

Figure S6. TEM-EDS pattern and elemental atomical analysis for Gel-CaP composite.

Table S3 Bound gelatin on Ti-TESPMA-Gel was measured by UV-Vis of FITC and the theoretical thickness of the swollen coatings was calculated based on the measured mass swelling degree.

Gel con.	Bound Gel(ug/cm^2)	Bound Gel Vol(/cm^2)	Thickness* (nm)
0.1%	15.22	1,1529*10^{-5}	502.65
1%	20.66	1,5653*10^{-5}	682.47
5%	30.94	2,3436*10^{-5}	1021.83
10%	21.81	1,6521*10^{-5}	720.3

*Swelling degree:4.3627

Table S4. Results of nanoindentation test for Ti and Ti-TESPMA-Gel-CaP.

samples	Nano-hardness (H,GPa)	Young's modulus (E, Gpa)	H/E
Ti	2.70±0.57	125.15±15.56	0.022
Ti-TESPMA-Gel-CaP	0.63±0.03	18.20±1.78	0.034

Table S5. Primer sequences used for qRT-PCR.

Primer	Forward(5' to 3')	Reserve(3' to 5')
BMP2	GGGACCCGCTGTCTTCTAGT	TCAACTCAAATTCGCTGAGGAC
BSP	CAGGGAGGCAGTGACTCTTC	AGTGTGGAAAGTGTGGCGTT
ALP	CCAACTCTTTTGTGCCAGAGA	GGCTACATTGGTGTTGAGCTTTT
OCN	CTGACCTCACAGATCCCAAGC	TGGTCTGATAGCTCGTCACAAG
OSX	GGAAAGGAGGCACAAAGAAGC	CCCCTTAGGCACTAGGAGC
RUNX2	AACGATCTGAGATTTGTGGGC	CCTGCGTGGGATTTCTTGGTT

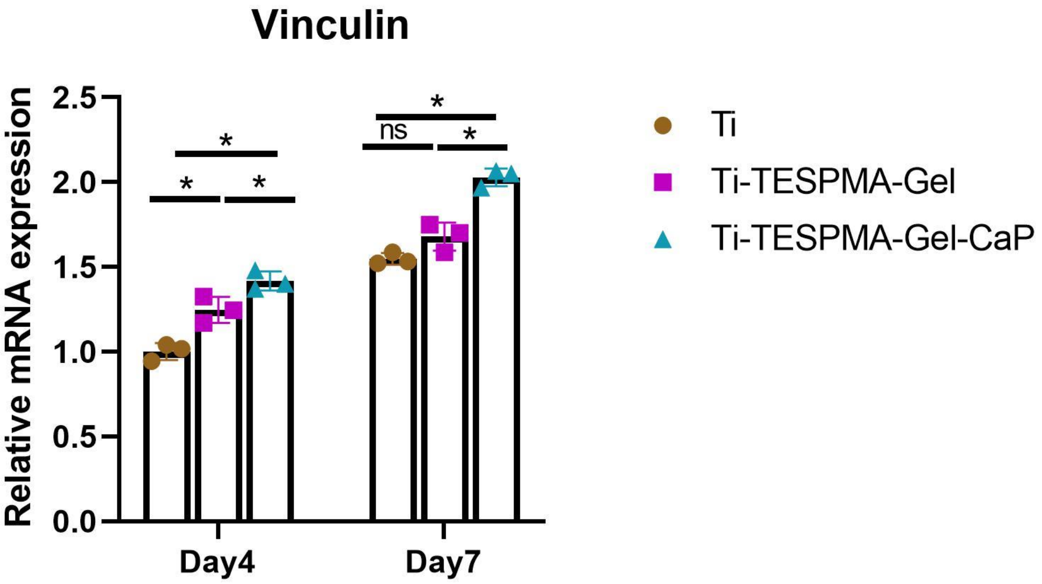

Figure S7. Real-time PCR analysis of Vinculin after culturing for 4 and 7 days on three groups. (*p<0.05)

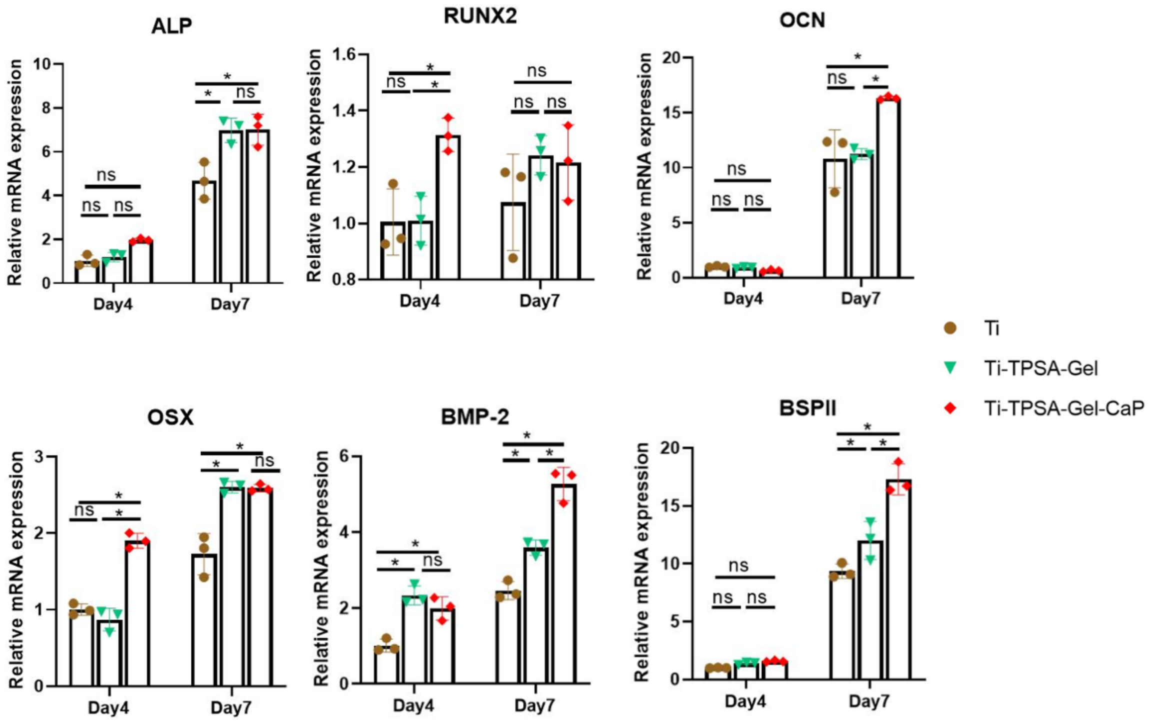

Figure S8. Real-time PCR analysis of osteogenic differentiation genes of MC3T3 cells after culturing for 4 and 7 days on Ti, Ti-TPSA-Gel, and Ti-TPSA-Gel-CaP. *p<0.05.

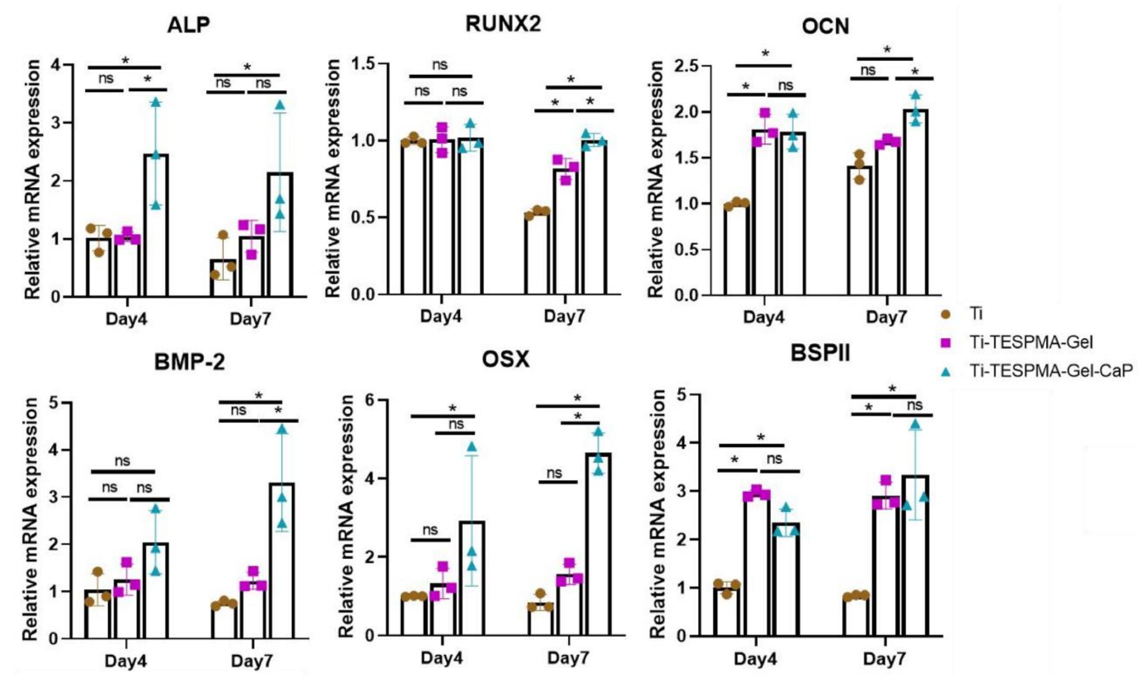

Figure S9. Real-time PCR analysis of osteogenic differentiation genes of BMSCs after culturing for 4 and 7 days on Ti, Ti-TESPMA-Gel, and Ti-TESPMA-Gel-CaP. *p<0.05.

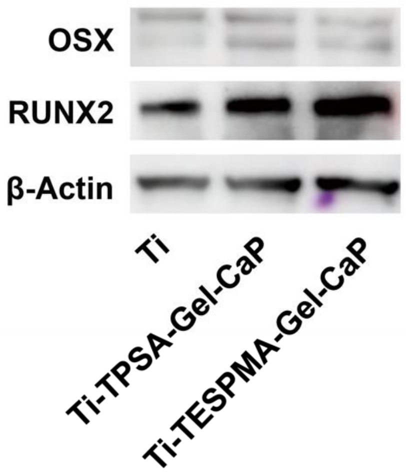

Figure S10. Western blot experiments showed the expression of OSX, RUNX2 proteins of BMSCs cultured for 72h on Ti, Ti-TPSA-Gel-CaP and Ti-TESPMA-Gel-CaP.
